# Global longitudinal strain detects subtle left ventricular systolic dysfunction in Duchenne muscular dystrophy patients and carriers

**DOI:** 10.1186/s43044-021-00214-0

**Published:** 2021-10-19

**Authors:** Mahmoud Shehta, Mona Mostafa Rayan, Nagia Aly Fahmy, Ahmed Onsy, Islam Bastawy

**Affiliations:** 1 Department of Cardiology, Ain Shams University, 38 Ramsis Street, El Abbaseya, Cairo, Egypt; 2 Department of Neurology, Ain Shams University, Cairo, Egypt

**Keywords:** Global longitudinal strain, Cardiomyopathy, Neuromuscular disorders, X-linked recessive, Heart failure

## Abstract

**Background:**

With the continuous improvement of the respiratory care of Duchenne muscular dystrophy patients, cardiac manifestations (heart failure and arrhythmias) become the leading causes of morbidity and mortality. Early identification of cardiac muscle affection is crucial to start anti-failure drugs that reverse remodeling and improve prognosis. This study aimed to detect subtle cardiac changes in Duchenne muscular dystrophy patients and carriers using electrocardiography and echocardiography.

**Results:**

This study included genetically diagnosed Duchenne muscular dystrophy patients (28 males) and carriers (25 females) and compared them to healthy gender-matched control groups. All study participants underwent clinical assessment, 12-lead electrocardiography, and global longitudinal strain augmented echocardiography. In the current study, Duchenne muscular dystrophy patients had higher heart rates, smaller left ventricular internal diameters, left atrial diameter, lower ejection fraction, and worse left ventricular global longitudinal strain in comparison with the control group. The global longitudinal strain inversely correlated with the age of Duchenne muscular dystrophy patients. The number of exon mutations did not affect electrocardiography and echocardiographic findings. Exon mutations 45–47 and 51–54 were significantly associated with an ejection fraction less than 60%. Duchenne muscular dystrophy carriers had smaller left ventricular wall diameters, left ventricular end-diastolic diameter, left atrial diameter, and worse left ventricular global longitudinal strain in comparison with the control group.

**Conclusions:**

Left ventricular global longitudinal strain could detect subtle left ventricular systolic dysfunction in Duchenne muscular dystrophy patients and carriers before the decline of left ventricular ejection fraction.

**Supplementary Information:**

The online version contains supplementary material available at 10.1186/s43044-021-00214-0.

## Background

Duchenne muscular dystrophy (DMD) is the most common inherited neuromuscular disorder that shows an X-linked recessive inheritance. Dystrophin gene mutations on chromosome Xp21 result in absent or deficient functional dystrophin protein in skeletal and cardiac muscles distorting their structure with subsequent fibrosis and weakness that impair their functions [1]. Respiratory and cardiac muscle weakness is considered the most common cause of morbidity and mortality in these patients [2]. With the continuous improvement of their respiratory care, cardiac manifestations (heart failure and arrhythmias) become the leading causes of morbidity and mortality. Cardiac manifestations may occur in some female carriers [3]. Early identification of cardiac muscle affection is crucial to start anti-failure drugs that reverse remodeling and improve prognosis. Cardiac magnetic resonance (CMR) imaging is the investigation of choice despite being technically demanding. Transthoracic echocardiography is a simple alternative that is more suitable for those younger than 6–7 years [4, 5]. Recent advances in echocardiography using speckle tracking echocardiography that assesses myocardial deformation provided an early identification of myocardial affection comparable to CMR. This study aimed to detect subtle cardiac changes in DMD patients and carriers using electrocardiography (ECG) and echocardiography.

## Methods

This is an observational cross-sectional case–control study in which we revised the records of the neuromuscular unit in a tertiary care university hospital. Forty patients with genetically diagnosed DMD without previous cardiac history were contacted to join the study between July 2019 and July 2020. The genetic diagnosis was done using multiplex ligation-dependent probe amplification (MLPA) in Centogene laboratories, Germany using SALSA MLPA probemix P034-B2/P035-B1 provided by MRC-Holland to test for deletion or duplication exon mutations of the DMD gene (Gene parts included in the messenger ribonucleic acid (mRNA) that are translated into proteins are known as exons). If the test was negative, then amplicon-based next-generation sequencing was performed in Centogene laboratories, Germany to detect other mutations such as intron mutation (introns are gene parts that do not have protein-coding information), non-sense mutation (point mutation the results in premature stop codon), or missense mutation (point mutation that results in changed amino acid) [6].

Among the contacted patients, only 30 patients showed interest to join the study, but 1 of them was excluded due to his marked chest wall deformity which prevented obtaining proper loops for echocardiographic examination. Another adult patient was excluded from the study as his age was above 18 years. DMD patients were enrolled in group A (number = 28 males), while healthy gender-matched participants were enrolled in control group B (number = 28 males) without a history of previous cardiac disease, hypertension, or diabetes mellitus. Mothers and female relatives of the patients included in group A who were genetically tested positive to be carriers of dystrophin gene mutations were enrolled in group C (number = 25 females), while healthy gender-matched participants were enrolled in control group D (number = 25 females). DMD carriers and controls were not hypertensive, diabetic and they did not have a previous cardiac history.

All participants were subjected to detailed clinical cardiac assessment including identification of any symptoms of heart failure or arrhythmias (dyspnea, orthopnea, paroxysmal nocturnal dyspnea, peripheral edema, palpitations, and syncope) and clinical examination including measuring arterial blood pressure, pulse examination, detection of peripheral edema, cardiac auscultation for heart sounds, and cardiac murmurs.

Three-channel 12-lead surface ECG was done for all the study participants using (CM 300 A, Comen, China) machine. Heart rate and heart rhythm were identified and the following measurements were taken. (a) PR interval: from the onset of the P wave to the onset of the QRS complex. (b) QRS complex duration: from the onset of the Q wave to the end of the S wave.

Standard ECG-gated trans-thoracic echocardiography was performed for all subjects using a General Electric (GE) S7 machine using an M4S matrix sector array probe having a frequency of 2.5 mega Hertz. Standard images were obtained at a depth of 16-cm (cm) in the parasternal (long- and short-axis views) and apical (2-, 3-, and 4-chamber images) views while subject in left lateral position. Standard ECG-gated 2-dimensional (2D) and color Doppler data were saved as loops. Motion (M)-mode, 2D, tissue Doppler imaging (TDI) as well as pulsed and continuous Doppler flow across the different heart valves in all the standard views were done according to the recommendations of the American Society of Echocardiography [7] to obtain the following: (a) Left ventricular (LV) ejection fraction (EF): Manual and semi-automated tracing was used to measure EF (by modified Simpson’s method) from apical 2- and 4-chamber views, (b) LV diastolic function parameters using mitral valve inflow pattern (E/A ratio, deceleration time, lateral e′, E/e′), (c) interventricular and posterior wall diameter at the end-diastole, (d) LV end-diastolic diameter (LVEDD) and LV end-systolic diameter (LVESD), (e) LV end-diastolic volume (LVEDV) and LV end-systolic volume (LVEDV), (f) left atrium (LA) diameter, (g) global longitudinal strain (GLS) was measured for all study participants using digital loops acquired from apical 2-, 3- and 4-chamber views. The recorded data were stored then analyzed independently through 2 experienced echocardiography consultants with the offline-workstation software Echo-PAC Dimension [12.0, GE Medical Systems GmbH, Germany] to calculate peak GLS. Using the automated function imaging software, a point-and-click approach was used to track the endocardial contour. The average LV GLS was measured automatically in all study participants.

### Statistical analysis

After data collection, revision and coding, it was entered into version 23 of Statistical Package for Social Science (IBM SPSS). Mean and standard deviation described parametric quantitative data while percentages and numbers were used to describe qualitative variables. Comparison between the study and control groups was done using the Chi-square test for qualitative data, and independent t-test was used for comparing quantitative data with parametric distribution between both groups. Spearman correlation coefficients were used to assess the correlation between the number of exons and different ECG and echocardiographic parameters. Also were used to assess the correlation between the age of patients and LV GLS. The setting for the confidence interval was 95% and the margin of error accepted was set to 5%, so the *p* value was considered significant if *p* < 0.05.

## Results

### Duchenne muscular dystrophy patients

**Fig. 1:**
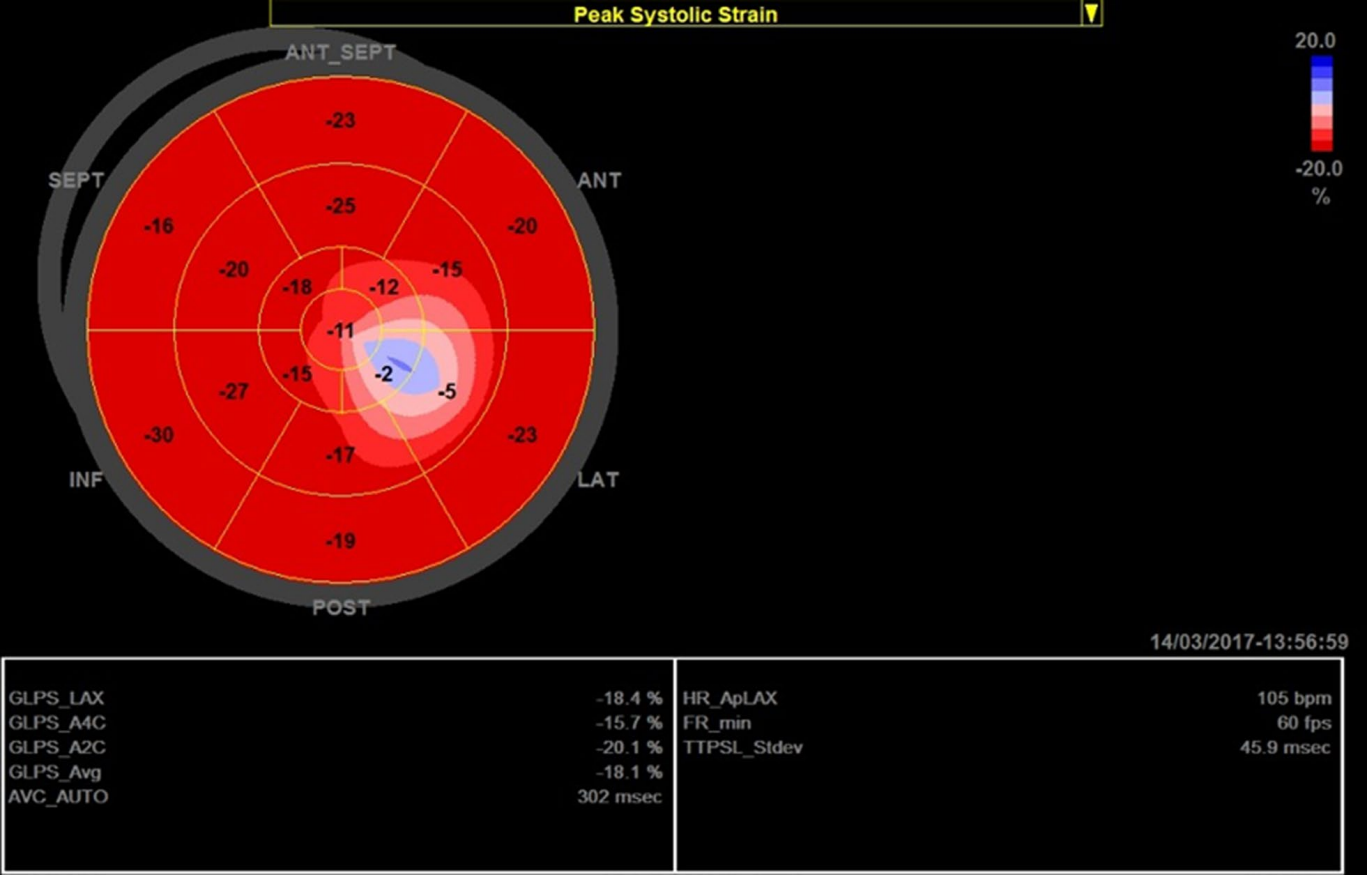
GLS of patient number 16 in group A

**Fig. 2:**
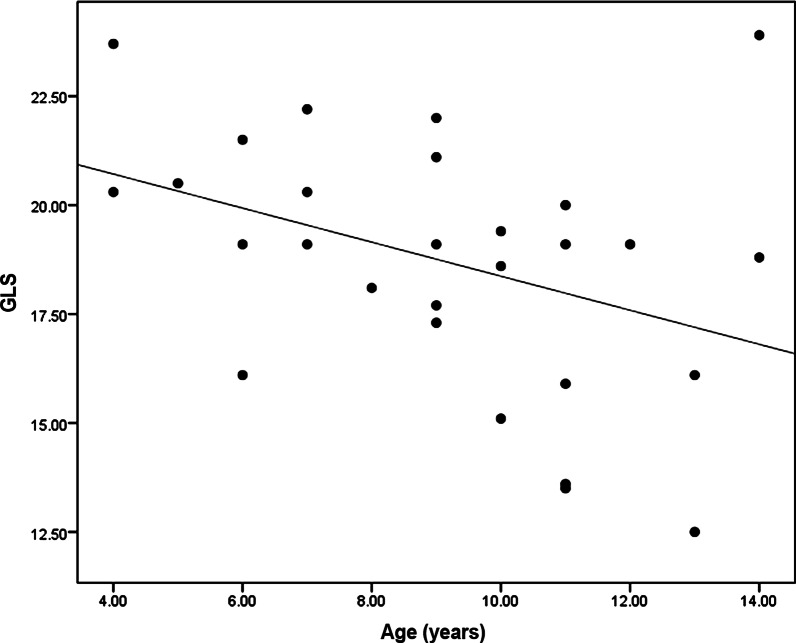
Correlation between age of DMD patients and GLS

On comparing ECG and echocardiographic findings between group A and B, group A had a smaller LVEDD, LVESD, LA diameter, higher heart rate, lower E/e' ratio, lower LV EF using modified Simpson’s method, and worse GLS compared to group B (Fig. 1). There was no significant difference between both groups on comparing LVEDV, LVESV, PR interval, and QRS complex duration (Table 1). There was a significant moderate inverse correlation between the age of patients and the GLS (*r* = − 0.4, *p* = 0.027) (Fig. 2).

**Table 1:** Comparing ECG and echocardiographic findings between group A and group B

	Group A (DMD patients)	Group B (Control)	*P* value
	(*n* = 28)	(*n* = 28)	
Age (years)	9.14 ± 2.85	10.64 ± 3.29	0.08
*Echocardiography*			
IVSd (mm)	6.21 ± 0.91	6.1 ± 0.99	0.66
PWd (mm)	6.46 ± 0.88	6 ± 0.98	0.07
LVEDD (mm)	37.5 ± 3.66	44.17 ± 4.38	**< 0.001**
LVESD (mm)	25.21 ± 2.69	27.71 ± 3.7	**0.005**
LVEDV (ml)	62.75 ± 13.71	66.71 ± 4.59	0.15
LVESV (ml)	23.14 ± 6.82	21.5 ± 3.38	0.132
LA dimension (mm)	27.35 ± 2.62	29.93 ± 3.62	**0.003**
EF% Modified Simpson's (%)	62.57 ± 5.38	67.64 ± 3.66	**< 0.001**
Diastolic function E/e'	4.5 ± 1.23	5.42 ± 0.96	**0.003**
GLS (%)	− 18.7 ± 2.91	− 21.25 ± 1.53	**< 0.001**
*ECG*			
HR (bpm)	95.75 ± 9.37	87.82 ± 7.01	**< 0.001**
PR interval (msec)	152.85 ± 16.91	153.46 ± 17.95	0.89
QRS duration (msec)	90 ± 5.93	87.5 ± 5.88	0.12

*bpm* bear per minute, *ECG* electrocardiography, *EF* ejection fraction, *GLS* global longitudinal strain, *HR* heart rate, *IVSd* interventricular septum diameter, *mm* millimeter, *LA* left atrium, *LVEDD* left ventricular end-diastolic diameter, *LVEDV* left ventricular end-diastolic volume, *LVESD* left ventricular end-systolic diameter, *LVESV* left ventricular end-systolic volume, *msec* millisecond, *PWd* posterior wall diameter

Genetic analysis of DMD patients showed that most of the patients had deletion or duplication exon mutations (25 patients with mutations involving one or more exons), 2 patients had a non-sense mutation, and 1 patient had an intron mutation. Most of these mutations were in the hot area (exon mutations 45–50) (Additional file 1: Table 1). Mutant exons 45–47 and 51–54 were significantly associated with EF < 60% (*p* = 0.045 and *p* = 0.022). The number of exon mutations was not associated with any significant difference in LV diastolic (*E/e*′, *p* = 0.316) or systolic (EF by modified Simpson’s method, *p* = 0.268) functions, GLS (= 0.885) PR interval (*p* = 0.763), and QRS duration (*p* = 0.580).

### Duchenne muscular dystrophy carriers

**Fig. 3:**
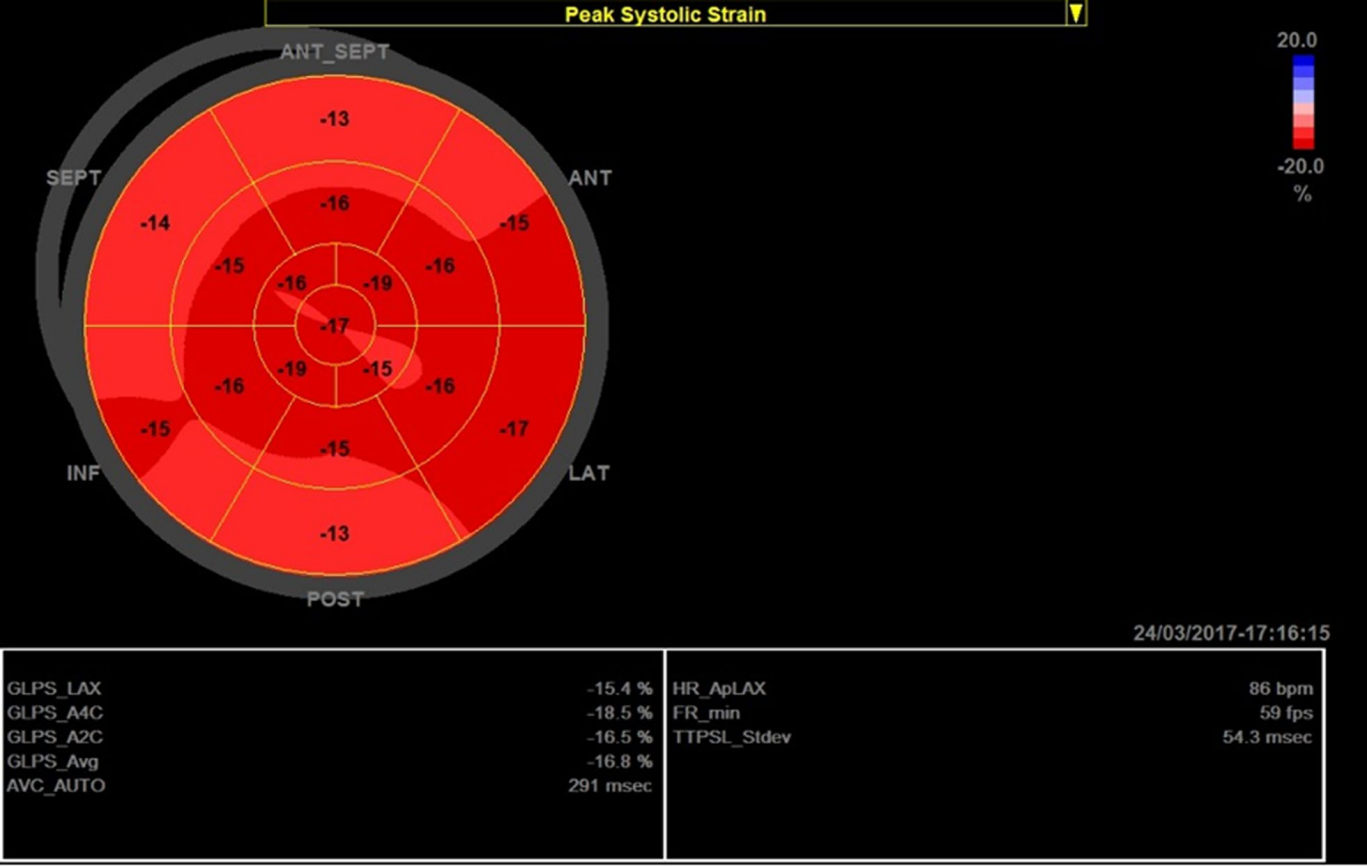
GLS of patient number 10 in group C

None of the female carriers was symptomatic. On comparing ECG and echocardiographic findings between group C and D, group C had smaller LV wall diameters, LVEDD, LA diameter, and worse GLS when compared to group D (Fig. 3). There was no significant difference between both groups on comparing LVESD, LVEDV, LVESV, LV EF using modified Simpson’s method, LV diastolic function by E/e', heart rate, PR interval, and QRS complex duration**.** There was no significant correlation between the age of carriers and the GLS (*r* = 0.013, *p* = 0.950)**.**

Genetic analysis of DMD carriers showed that most of the carriers had deletion or duplication exon mutations (22 carriers with mutations involving one or more exons), 2 carriers had a non-sense mutation, and 1 carrier had an intron mutation (Additional file 1: Table 2). The number of exon mutations was not associated with any significant difference in LV diastolic (*E/e*′, *p* = 0.525), or systolic functions (EF by modified Simpson’s method, *p* = 0.347), GLS (*p* = 0.448), PR interval (*p* = 0.536), and QRS duration (*p* = 0.731) (Table 2).

**Table 2:** Comparing ECG and echocardiographic findings between group C and group D

	Group C (female carriers)	Group D (control)	*p* value
	*n* = 25	*n* = 25	
Age (years)	30.28 ± 9.69	33.52 ± 6.63	0.17
*Echocardiography*			
IVSd (mm)	7.68 ± 1.38	9.64 ± 1.11	**< 0.001**
PWd (mm)	7.48 ± 1.45	9.32 ± 0.99	**< 0.001**
LVEDD (mm)	44.92 ± 4.09	48.44 ± 3.08	**0.001**
LVESD (mm)	29.48 ± 3.34	30.84 ± 2.15	0.09
LVED vol (ml)	93.64 ± 19.51	93.16 ± 18.44	0.93
LVES Vol (ml)	33.48 ± 9.95	36.76 ± 10.39	0.26
LA dimension (mm)	33.04 ± 4.32	35.64 ± 3.30	**0.02**
EF% Modified Simpson's (%)	63.84 ± 5.24	62.36 ± 5.30	0.32
Diastolic function E/E'	5.64 ± 1.04	5.18 ± 1.08	0.13
GLS	− 16.64 ± 3.55	− 21.12 ± 1.36	**< 0.001**
*ECG*			
HR (bpm)	81.16 ± 10.73	81.28 ± 7.65	0.96
PR interval (msec)	154.8 ± 18.11	146 ± 15.94	**0.05**
QRS duration (msec)	88.96 ± 7.10	89.52 ± 5.99	0.764

*bpm* bear per minute, *ECG* electrocardiography, *EF* ejection fraction, *GLS* global longitudinal strain, *HR* heart rate, *IVSd* interventricular septum diameter, *mm* millimeter, *LA* left atrium, *LVEDD* left ventricular end-diastolic diameter, *LVEDV* left ventricular end-diastolic volume, *LVESD* left ventricular end-systolic diameter, *LVESV* left ventricular end-systolic volume, *msec* millisecond, *PWd* posterior wall diameter

### Intra-observer and inter-observer variability

Strain analysis was done by 2 independent experts in echocardiography with acceptable intra-observer variability (0.93) and inter-observer variability (0.89).

## Discussion

Patients with inherited neuromuscular disorders have a lot of obstacles in receiving sufficient health care services in developing countries. This study aimed to early detect the decline in LV systolic functions before the decline in LV EF or the occurrence of heart failure symptoms in DMD patients or carriers who had visited the neuromuscular unit in the neurology department in a tertiary care university hospital using clinical assessment, ECG, and transthoracic echocardiography that was augmented by speckle tracking measurement of the LV GLS rather than CMR. The mean age of DMD patients was 9.14 ± 2.85 years, and 32% of patients were ≤ 7 years in which lack of co-operation might hinder undergoing CMR without anesthesia [4]. Cardiomyopathy is considered a leading cause of mortality in these patients and many studies showed that the majority of DMD after the third decade of their age have established cardiomyopathy [8]. Due to relative physical inactivity, clinically overt heart failure may be delayed, and myocardial damage precedes the decline in LV systolic function.

Group A patients showed a significantly higher rate as compared to age-matched controls. This is supported by several studies that found a lack of normal age-related heart rate decline in patients with DMD and postulated that the elevated resting heart rate may be a form of abnormal heart rate variability implicating autonomic dysfunction that is associated with myocardial fibrosis [9].

DMD patients and carriers had smaller LV dimensions as compared to controls. This may be attributed to myocardial atrophy and fibrosis of the LV in DMD patients and carriers documented by CMR using late gadolinium enhancement [10] and may be related to smaller body surface area and smaller body mass indices in DMD patients [11, 12]. Patchy myocardial fibrosis results in compensatory hypertrophy in healthy muscle fibers that increases LV mass initially before the decline of EF; however, with the disease progression, extensive fibrosis results in LV dilatation and dilated cardiomyopathy.

We observed that despite normal mean LV EF in DMD patients and carriers, the mean GLS was significantly lower in DMD patients and female carriers that indicated subclinical myocardial affection. This is supported by many studies that found that the application of myocardial strain imaging in DMD patients was characterized by decreased peak systolic strain, despite normal standard echocardiographic findings [11–14]. In mutation carriers, CMR revealed a pattern of fibrosis similar to that observed in DMD, even in the absence of overt muscular disease [15]. Normal LV GLS is considered less than − 18% [16], so despite being asymptomatic and having normal LV EF, 36% of DMD had abnormal LV GLS (10 patients) while 60% of female carriers had abnormal LV GLS (15 patients). The advantage of GLS is its ability to detect subtle LV dysfunction even before the onset of symptoms or the decline of LV EF [17] that allows early initiation of anti-failure measures. There is still a debate on the proper time to start anti-failure drugs. However, early treatment is associated with better outcomes [18].

The genetic diagnosis was mandatory for enrolling patients in this study. The most common prevalent exon mutations were from 45 to 50 that is matching with previous studies [19]. In this study exon mutations, 45–47 and 51–54 were significantly associated with EF < 60%. Variable exon mutations were previously related to cardiomyopathy; however, these results may be misleading due to the small number of patients with the same exon mutations [19, 20]. In the present study, the number of exon mutations was not correlated with LV systolic or diastolic functions. This finding is supported by Ashwath and her colleagues who found no relation between the number of exon mutations and the incidence or severity of cardiomyopathy [21].

Moreover, a significant moderate inverse correlation was found between the age of DMD patients and the decline in GLS. This is supported by Nigro et al. and Cirino et al. who found that cardiac problems typically start as latent cardiomyopathy without symptoms evolving into clinically overt cardiac disease in 14% of patients under the age of 14 years, to be present in 57% of patients over 18 years of age [19, 22]. This study presents global longitudinal strain as a simple potential tool that could be used in the periodic follow-up of patients and carriers with a lower cost in comparison with CMR to detect subtle LV systolic dysfunction that may allow early initiation of anti-failure treatments that improve their prognosis.

### Limitations

It is a single-center study that included a relatively small number of patients and didn’t include CMR which is the gold standard for diagnosing cardiomyopathy in DMD patients and carriers.

## Conclusions

This study presented the value of early cardiac surveillance not only in DMD patients but also in female carriers even before the onset of cardiac symptoms using relatively simple techniques. The assessment of GLS was able to detect subtle LV systolic dysfunction in patients and carriers before the decline of EF.

## Supplementary Information

**Additional file 1**: **Table S1**. Sites of exon mutation in Group A. **Table S2**. Different sites of exon mutation in group C.

## Data Availability

The datasets used and analyzed during the current study are available from the corresponding author on reasonable request.

## Abbreviations

2D: 2-Dimensional
bpm: Beat per minute.
cm: Centimeter
CMR: Cardiac magnetic resonance
DMD: Duchenne muscular dystrophy
ECG: Electrocardiogram
EF: Ejection fraction
GE: General Electric
GLS: Global longitudinal strain
IVSd: Interventricular septum diameter at the end-diastole
LA: Left atrium diameter
LV: Left ventricle
LVEDD: LV end-diastolic diameter
LVEDV: LV end-diastolic volume
LVESD: LV end-systolic diameter
LVESV: LV end-systolic volume
MLPA: Multiplex ligation-dependent probe amplification
mm: Millimeter
M-mode: Motion mode
mRNA: Messenger ribonucleic acid
msec: Millisecond
Pwd: Posterior wall diameter at the end-diastole
SPSS: Statistical Package for Social Science
TDI: Tissue Doppler imaging
