# Supplementary material for: Global longitudinal strain detects subtle left ventricular systolic dysfunction in Duchenne muscular dystrophy patients and carriers
Source: Egypt Heart J. 2021 Oct 19;73:91. doi: 10.1186/s43044-021-00214-0 (PMC8526669; doi:10.1186/s43044-021-00214-0)
Supplement: Supplementary file 1 — Additional file 1: Table S1. Sites of exon mutation in Group A. Table S2. Different sites of exon mutation in group C. [file 43044_2021_214_MOESM1_ESM.docx]

Table (1): Sites of exon mutation in Group (A)

| **Exon mutation** | **Patients** | |
| --- | --- | --- |
|  | **No.** | **%** |
| 1 | 2 | 6.9% |
| 2 | 4 | 13.8% |
| 3 | 3 | 10.3% |
| 4 | 3 | 10.3% |
| 5 | 3 | 10.3% |
| 6 | 3 | 10.3% |
| 7 | 1 | 3.4% |
| 8 | 2 | 6.9% |
| 9 | 2 | 6.9% |
| 10 | 2 | 6.9% |
| 11 | 2 | 6.9% |
| 12 | 2 | 6.9% |
| 13 | 2 | 6.9% |
| 14 | 2 | 6.9% |
| 15 | 2 | 6.9% |
| 16 | 2 | 6.9% |
| 17 | 2 | 6.9% |
| 18 | 2 | 6.9% |
| 19 | 2 | 6.9% |
| 20 | 2 | 6.9% |
| 21 | 2 | 6.9% |
| 41 | 2 | 6.9% |
| 43 | 1 | 3.4% |
| 45 | 7 | 24.1% |
| 46 | 7 | 24.1% |
| 47 | 7 | 24.1% |
| 48 | 8 | 27.6% |
| 49 | 11 | 37.9% |
| 50 | 11 | 37.9% |
| 51 | 4 | 13.8% |
| 52 | 4 | 13.8% |
| 53 | 4 | 13.8% |
| 54 | 4 | 13.8% |
| 55 | 3 | 10.3% |
| 56 | 1 | 3.4% |
| 57 | 1 | 3.4% |
| 58 | 1 | 3.4% |
| 59 | 1 | 3.4% |
| 60 | 1 | 3.4% |
| 61 | 1 | 3.4% |
| 62 | 1 | 3.4% |
| 65 | 3 | 10.3% |

Table (2) Different sites of exon mutation in group C:

| **Exon mutation** | **Carriers** | |
| --- | --- | --- |
|  | **No.** | **%** |
| 1 | 1 | 4.0% |
| 2 | 2 | 8.0% |
| 3 | 2 | 8.0% |
| 4 | 2 | 8.0% |
| 5 | 2 | 8.0% |
| 6 | 2 | 8.0% |
| 7 | 1 | 4.0% |
| 8 | 1 | 4.0% |
| 9 | 1 | 4.0% |
| 10 | 1 | 4.0% |
| 11 | 1 | 4.0% |
| 12 | 1 | 4.0% |
| 13 | 1 | 4.0% |
| 14 | 1 | 4.0% |
| 15 | 1 | 4.0% |
| 16 | 1 | 4.0% |
| 17 | 1 | 4.0% |
| 18 | 1 | 4.0% |
| 19 | 1 | 4.0% |
| 20 | 1 | 4.0% |
| 21 | 1 | 4.0% |
| 41 | 2 | 8.0% |
| 43 | 1 | 4.0% |
| 45 | 6 | 24.0% |
| 46 | 6 | 24.0% |
| 47 | 6 | 24.0% |
| 48 | 7 | 28.0% |
| 49 | 10 | 40.0% |
| 50 | 10 | 40.0% |
| 51 | 5 | 20.0% |
| 52 | 5 | 20.0% |
| 53 | 5 | 20.0% |
| 54 | 5 | 20.0% |
| 55 | 2 | 8.0% |
| 56 | 1 | 4.0% |
| 57 | 1 | 4.0% |
| 58 | 1 | 4.0% |
| 59 | 1 | 4.0% |
| 60 | 1 | 4.0% |
| 61 | 1 | 4.0% |
| 62 | 1 | 4.0% |
| 65 | 4 | 16.0% |
